# Health Tract, No. 43

**Published:** 1862-02

**Authors:** 


HEALTH TRACT, No. 43.

From Hall's Journal of Health, $1 a year, New-York.

### PREMONITIONS.

An incalculable amount of sickness, suffering, and premature death would be avoided every year, if we could be induced to heed the warnings, the premonitions, which kindly nature gives of the coming on of the great enemy, disease. Many a mother especially, has lost a darling child, to her life-long sorrow, by failing to observe the approach of disease, in some unusual act or circumstance connected with her offspring.

1. If an adult or child wakes up thirsty in the morning, however apparently well at the moment, or the preceding evening, there will be illness before noon always, infallibly. It is generally averted by remaining warm in bed, in a cool, well-ventilated room, eating nothing, but drinking plentifully of some hot tea all day; some little may be eaten in the afternoon by a child. But as long as a person wakes with thirst in the morning, there is an absence of health—there is fever.

2. If, when not habitual to him, one is waked up early in the morning by an inclination to stool, especially if there is a feeling of debility afterwards, it is the premonition of diarrhea, summer complaint, dysentery, or cholera. There should be perfect quietude, etc., as above; in addition, a piece of warm, thick, woolen flannel should be wrapped tightly around the abdomen, (belly;) the drink should be boiled milk; or far better, eat pieces of ice all the time, and thus keep the thirst perfectly subdued; eat nothing but boiled rice, corn starch, sago, or tapioca, and continue all these until the tiredness and thirst are gone, the strength returned, and the bowels have been quiet for twelve hours, returning slowly to the usual activities and diet.

3. If a child is silent, or hangs around its mother to lay its head on her lap, or is most unusually fretful, or takes no interest in its former amusements, except for a fitful moment at a time, it is certainly sick, and not slightly so. Send at once for a physician, for you can't tell where or in what form the malady will break out; and in children especially, you can never tell where any particular ailment will end.

4. When there is little or no appetite for breakfast, the contrary having been the case, the child is sick, and should be put to bed, drinking nothing but warm teas, eating not an atom until noon, then act according to developments.

5. If a child manifests a most unusual heartiness for supper, for several nights in succession, it will certainly be sick within a week, unless controlled.

6. If there is an instantaneous sensation of sickness at stomach, during a meal, eat not a particle more; if just before a meal, omit it; if after a meal, go out of doors, and keep out in active exercise for several hours, and omit the next meal, for all these things indicate an excess of blood or bile, and exercise should be taken to work it off, and abstinence, to cut off an additional supply, until the healthful equilibrium is restored.

7. A kind of glimmer before the eyes, making reading or sewing an effort, however well you may feel, will certainly be followed by head-ache or other discomfort, for there is too much blood, or it is impure; exercise it off in the open air, and omit a meal or two.

8. If you are not called to stool at the accustomed hour, (except when traveling, then let things take care of themselves—do nothing,) eat not an atom until it is done, for loss of appetite, or nausea, or loose bowels, or biliousness, is certainly impending. Exercise freely out of doors, and drink cold water or hot teas to the fullest desired extent.

9. If there is a most unnatural indisposition to exertion, you need rest, quiet, and abstinence; exercise in weariness never does any good, always harm. But if causelessly despondent, or there is a general feeling of discomfort, the blood is bad, warm the feet, unload the bowels, eat nothing for twelve hours, and be out of doors all day.

10. If, without any known cause, or special pain, you are exceedingly restless, can not sleep, or if you do, it is dreamy, disturbed, or distressing, you have eaten too much, or are on the verge of some illness. Take nothing next day but hot drinks and toasted bread, and a plenty of out-door exercise. In all these cases, a thorough washing with soap and hot water, and vigorous bodily friction, greatly expedite restoration.

